# Predictors and Outcomes of Airway Management in Patients Presenting to the Emergency Department With Overdose and Decreased Consciousness: A Scoping Review

**DOI:** 10.1155/emmi/8071582

**Published:** 2025-10-04

**Authors:** Richard A. F. Pellatt, Sarah Ishak, Justin Clark, Katherine Isoardi, Robert S. Ware, Gerben Keijzers

**Affiliations:** ^1^Emergency Department, Gold Coast University Hospital, 1 Hospital Boulevard, Gold Coast 4215, Queensland, Australia; ^2^School of Medicine and Dentistry, Griffith University, Parklands Drive, Southport, Gold Coast Campus, Gold Coast 4222, Queensland, Australia; ^3^Faculty of Health Sciences and Medicine, Bond University, 14 University Drive, Robina, Gold Coast 4226, Queensland, Australia; ^4^LifeFlight Retrieval Medicine, LifeFlight Australia, Brisbane, Queensland, Australia; ^5^Royal Prince Alfred Hospital, 50 Missenden Road, Camperdown, New South Wales, Australia; ^6^Institute for Evidence-Based Healthcare, Bond University, Gold Coast, Australia; ^7^Clinical Toxicology Unit, Princess Alexandra Hospital, Brisbane, Australia; ^8^Faculty of Medicine, University of Queensland, Brisbane, Australia; ^9^Griffith Biostatistics Unit, Griffith University, Parklands Drive, Southport, Gold Coast Campus, Gold Coast 4222, Queensland, Australia

**Keywords:** emergency medicine, endotracheal intubation, Glasgow Coma Scale, overdose, poisoned, toxicology

## Abstract

**Objective:**

To explore evidence relating to the airway management (ranging from observation to intubation) of patients presenting to the emergency department with drug overdose and decreased consciousness.

**Introduction:**

Recreational and prescription drug overdoses are a common reason for patients to present to the emergency department. Patients often have a reduced level of consciousness due to the effects of the drugs involved. There are no evidence-based guidelines identifying which patients can be safely observed, compared with those requiring advanced airway management such as intubation.

**Inclusion Criteria:**

Adult patients presenting to the emergency department with drug overdose (recreational drugs, e.g., gamma-hydroxybutyrate and alcohol, and prescription drugs, e.g., benzodiazepines and opioids). Management strategies from intubation (e.g., rapid sequence intubation, emergency airway management) to observation/no intubation (e.g., high flow nasal oxygen, observation, monitoring, recovery position) were included. Studies are needed to describe an approach to airway management in overdose patients with decreased consciousness, which included all primary research. Outcomes included predictors of intubation, mortality, hospital admission, length of stay and complications.

**Methods:**

With the assistance of a Systematic Review Accelerator, we searched PubMed, Cochrane CENTRAL and Embase. Databases were searched from inception to 2^nd^ April 2024, with no publication language restrictions. We additionally conducted a backward and forward citation search.

**Results:**

Forty-five studies were included (one randomised controlled trial, 14 prospective observational studies, 25 retrospective observational studies and five with other methodology). Four major narrative themes were identified: (1) risk stratifying the decision to intubate; (2) noninvasive monitoring of the patient with overdose; (3) scoring systems predicting the need to intubate; (4) targeted gamma-hydroxybutyrate-specific literature.

**Conclusions:**

Literature on the airway management of emergency department patients with overdose and decreased level of consciousness provides mostly low-quality evidence, with only one RCT. Further higher-level research and evidence are required.

## 1. Introduction

Patients commonly present to the emergency department (ED) with drug overdose. The toxicological effects of these drugs can include reduced consciousness and coma. Currently, there is a lack of high-quality evidence on the best approach to the airway management of these patients [1, 2]. Management options include observation, basic airway management such as airway manoeuvres and applying oxygen, to more advanced techniques including intubation and mechanical ventilation.

High-quality research is scarce for several reasons. As patients have a decreased level of consciousness and awareness, obtaining accurate information can be difficult [3]. Securing a homogeneous patient subgroup is challenging due to the large number of potential drugs involved and the differences in their clinical presentation, course and management. This, combined with the inability to provide a history and details regarding drug types and amounts, makes curating a controlled study population challenging. As a result of this heterogeneity, guidelines and recommended approaches are often based on expert opinion [1]. Consequently, airway management is highly variable due to patient features, clinician experience, geographical location and available hospital services.

As such, we identified the need to conduct a scoping review to assess the available evidence regarding airway management in this often undifferentiated patient group. The aim of this review was to explore the evidence in relation to the predictors and outcomes of airway management of patients presenting to the ED with drug overdose and decreased consciousness and associated patient outcomes.

## 2. Methods

### 2.1. Protocol and Registration

We conducted a scoping review of the evidence concerning airway management of the ED patient presenting with overdose and decreased level of consciousness, in particular, predictors and outcomes of airway management in this population. The review was conducted in accordance with guidance from the Joanna Briggs Institute [4] and reported following the Preferred Reporting Items for Systematic Reviews and Meta-Analyses Extension for Scoping Reviews guidelines (PRISMA-ScR) [5]. The protocol for this review is registered on Open Science Framework (OSF) [6].

### 2.2. Search Strategy

An initial search was designed and run in PubMed by an experienced Cochrane Information Specialist and Health Librarian (JC). That search was translated using the Polyglot Search Translator to search Cochrane CENTRAL (via Wiley) and Embase (via Elsevier) [7]. The searches were run on the 2 April 2024, and no publication or date restrictions were applied to the search. Full search strings for all databases are available in Appendix 1. A backward and forward citation search was conducted using the SpiderCite tool available via the Systematic Review Accelerator (SRA) [7].

### 2.3. Selection Criteria

We included all primary literature comprised of randomised controlled trials (RCTs), observational studies, prospective and retrospective cohort studies, case–control studies, analytical cross-sectional studies, descriptive cross-sectional studies, case series, individual case reports and qualitative studies. To be included, the target population of studies needed to be adults requiring airway management when presenting to the ED with drug overdose (recreational drugs, e.g., gamma-hydroxybutyrate and alcohol, as well as prescription drugs, e.g., benzodiazepines). Paediatric patients with overdose were excluded. Existing reviews were excluded.

Management strategies from intubation (e.g., rapid sequence intubation, emergency airway management) to observation/no intubation (e.g., high flow nasal oxygen, observation, monitoring, recovery position) were included. Studies are needed to describe an approach to airway management in overdose patients with decreased consciousness, which included all primary research. Outcomes included predictors of intubation, mortality, hospital admission, length of stay (LoS) and complications.

### 2.4. Study Selection

All identified citations were collated and uploaded to the SRA Screenatron tool, and titles and abstracts were screened independently by two reviewers (Richard A. F. Pellatt and Sarah Ishak) against the inclusion criteria for the review. Disagreements between reviewers at each stage of the selection process were compared using the SRA Disputatron and were resolved through consensus [7].

### 2.5. Data Extraction and Synthesis

A data extraction form was developed. Data were extracted by two reviewers (Richard A. F. Pellatt and Sarah Ishak) independently, any disagreements were resolved through discussion. The data extracted included specific details about the participants, concept, context, study methods and key findings.

## 3. Results

### 3.1. Results

#### 3.1.1. Study Selection and Characteristics

**Figure 1:**
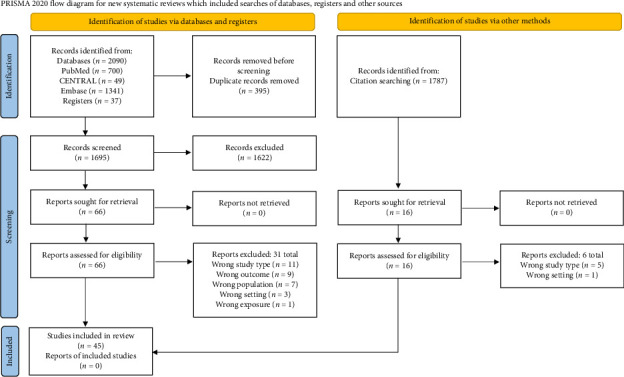
PRISMA flow diagram.

The database search resulted in a total of 2090 records, after removing duplicates, 1695 records remained for screening. A total of 66 reports were retrieved for full-text screening. A further 1787 records were identified via forward and backward citation searching, of which 16 were assessed for eligibility. Overall, 45 studies were included in the review.  Figure 1 demonstrates the flow diagram of included studies. A full list of excluded studies is included in Appendix 2.

**Figure 2:**
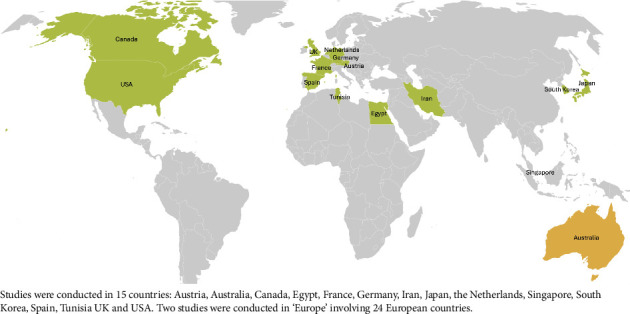
Map showing countries where included studies were conducted.

Studies were conducted in 15 countries: Austria, Australia, Canada, Egypt, France, Germany, Iran, Japan, the Netherlands, Singapore, South Korea, Spain, Tunisia, the United Kingdom (UK) and the United States of America (USA) (Figure 2). Two studies specified that they were conducted in Europe. Forty-three were written in English; one study had an English abstract and full text in German; one study was written in Spanish. The year of publication ranged from 1993 to 2023. Most studies were published after 2010 (*n* = 33; 70%).  Table 1 summarises the key characteristics of included studies.  Table 2 summarises the main outcomes of the included studies.

#### 3.1.2. Age

An Australian study examined 800 patients with overdose, of whom 78 were admitted to intensive care unit (ICU) (56 intubated), and found that patients admitted to ICU were older (median age 41.4 vs. 36.9 years) than those who were not [40]. A Dutch retrospective cohort study of 9679 ICU admissions for patients with overdose found age over 55 to independently predict the need for ICU [33]. Another Dutch study of 255 overdose patients found that high acuity patients were older than low acuity patients (mean age 40 vs. 35 years) [32]. Nagashima et al. observed similar findings in 132 intubated vs nonintubated patients with overdose in a Japanese study [43]. In a large case–control study, Noseda et al. found age > 35 was associated with admission to critical care [46]. Conversely, Hua et al. found younger age a predictor of the need for intubation in overdose in 2497 patients presenting with overdose in a secondary data analysis from a prospective cohort study in the USA [2]. The average age in the 87 (3.5%) intubated patients was 41 compared to 45 in those who were not intubated (mean difference −3.6 years, 95% CI −7.4 to −0.2). Other studies found no difference in age for intubated and nonintubated patients [18, 27]. Most studies suggest that older age predicts the need for intensive care and therefore intubation.

#### 3.1.3. Sex

In general, males were overrepresented compared with females across studies [29, 41, 42, 45–48]. In a large airway registry study in the USA, Kunzler et al. found males represented 66% of 1983 intubated patients with overdose [42]. Eizadi-Mood et al. found males were more likely to have fatal poisoning in a cross-sectional study conducted in Iran [41]. In a longitudinal analysis conducted in European EDs, Miró et al. found males were significantly more likely to be intubated in recreational drug overdoses; ICU admission and death were similar by sex [48]. In determining admission to critical care following acute recreational drug toxicity in a retrospective multicentre matched case–control study, Noseda et al. did not find that male sex was associated with critical care admission [46].

#### 3.1.4. Comorbidities

A prior history of obstructive lung disease was associated with a higher likelihood of endotracheal intubation in the analysis by Hua et al. [2]. In their cohort, 87 (3.5%) of 2497 patients with drug overdose were intubated. Obstructive lung disease was an independent clinical risk factor for intubation (OR 6.6, 95% CI 3.5–12.3). Patients with a history of obstructive lung disease had a higher likelihood of requiring endotracheal intubation [49]. In a retrospective review of 800 patients presenting to ED in Australia following drug overdose, Savage et al. found that a prior history of drug overdose and a psychiatric history were predictors for ICU admission, usually due to a need for endotracheal intubation [40]. Brandenburg et al. found cirrhosis and chronic respiratory disease predicted ICU admission (± intubation) [33].

#### 3.1.5. Drugs Involved

In an analysis of 2724 intubated overdose patients in the American College of Medical Toxicology registry, Beauchamp et al. found the most common single substance exposures were sedative hypnotics (*n* = 266, 9.8%), antidepressants (*n* = 236, 8.7%) and opioids (*n* = 217, 8.0%) [29]. Donald et al. observed a higher proportion of tricyclic antidepressant and cardiotoxic drugs in 12 intubated patients, compared with alcohol or sedatives in 14 nonintubated patients in a small prospective parallel group comparison study in the UK [18]. In an observation study involving 1421 overdose patients in Singapore, Kant et al. observed that psychiatric and benzodiazepine medications were more commonly involved in the 121 patients admitted to the ICU, of whom 56 were intubated [20]. In multivariable analysis, Savage et al. found atypical antipsychotics were more likely to be implicated in ICU admission (and intubation) (OR 1.96, 95% CI 1.12–3.41) [40]. Nagashima et al. found an association between antipsychotics (OR 2.27, 95% CI 1.07–4.83) and antidepressants (OR 2.50, 95% CI 1.14–5.64) with tracheal intubation that persisted on multivariable analysis [43].

#### 3.1.6. Polypharmacy

Several studies noted that polypharmacy overdoses were more likely to require ICU-level care (and intubation) [9, 14, 27]. When establishing a prediction model for intoxicated patients requiring admission to the ICU, Brandenburg et al. found that alcohol as an ingestant predicted ICU admission to be unlikely [33]. Of 1160 patients with alcohol intoxication, only 44 (3.8%) needed ICU admission. Noseda et al. also linked polypharmacy to critical care admission [46].

#### 3.1.7. Aspiration Risk

Kunzler et al. examined a large airway registry of intubated patients in the USA, comparing intubations for drug overdose with other indications [42]. They reported adverse events, rescue surgical airways and intubation success. Intubation at the first attempt was comparable for patients with overdose, compared with all other indications (90 vs. 89%). There were fewer instances of hypotension, fewer adverse events and no surgical airways in the overdose group. They concluded that intubation was less risky for the indication of overdose compared to other indications for intubation. However, the baseline characteristics of the two groups differed significantly, and the drugs involved were not recorded.

#### 3.1.8. Level of Consciousness

There is conflicting evidence to support the use of the Glasgow Coma Scale (GCS) in a toxicology setting and outside of trauma [8, 12, 18, 50]. Some literature supports the notion that patients with GCS < 8 require endotracheal intubation [8, 10, 12, 23, 24, 27, 31, 33, 43]. However, data also demonstrate it is safe to manage overdose patients with a decreased GCS conservatively [18, 19, 36, 37, 49].

In a prospective parallel group comparison study, Donald et al. noted that four patients with a GCS of three were safely managed without intubation, using an oropharyngeal airway [18]. The study examined 26 patients presenting with drug overdose and reduced consciousness (GCS of less than eight). Twelve were intubated and 14 managed without intubation. There were no adverse outcomes for the nonintubated group, who had overall shorter length of hospital stay, and received fewer computed tomography (CT) brain scans [18]. In a retrospective chart review by Mehrl et al., GCS did not correlate with aspiration risk [37]. In patients with a GCS of eight or less, 2/45 (4%) nonintubated patients developed aspiration pneumonitis, compared to 2/8 (25%) intubated patients [37]. In an observational study of 73 patients with reduced consciousness due to drug or alcohol intoxication, Duncan and Thakore noted that 12 patients had a GCS of eight or less on admission, and none required intubation [19].

In the only RCT identified concerning drug overdose with reduced consciousness, Freund et al. randomised 225 patients with a GCS of eight or less between standard care (intubation) and an interventional strategy of withholding intubation [45]. Fewer patients were intubated in the intervention group. The interventional group received mechanical ventilation less often (18.1% vs. 59.6%). No patients died in either group. The hierarchical composite end point of death, length of ICU stay and length of hospital stay was improved in the intervention group with a ratio of 1.85 (95% CI 1.33–2.58; *p* < 0.01). The authors concluded that withholding intubation was safe. There was a higher rate of pneumonia in the intubation group.

Conversely, several studies showed GCS can predict intubation (or ICU admission and by proxy intubation). Sabzghabaee et al. found GCS to be a good discriminator for admission to ICU and adverse patient outcomes in a prospective follow-up study conducted in an Iranian ED [23]. Van Den Berg et al. and Hamad et al. observed similar findings [10, 31]. In a prospective observational study of 414 overdose patients, Chan et al. found that a GCS of less than eight predicted intubation [8]. Similarly, in a retrospective cohort study of 9679 overdose patients, Brandenburg et al. showed a GCS of less than six to be predictive of ICU admission [33]. Nagashima et al. also found a low GCS to be predictive of intubation [43].

The association between GCS in overdose patients and clinical actions was examined in other ways. In a prospective, observational study, Adnet et al. found that a lower GCS predicted a more difficult intubation [15]. They proposed that this group had less frequent use of sedatives or RSI, making intubation more difficult. Kelly et al. showed that the AVPU scale correlated with GCS in the assessment of conscious level in patients with overdose [13].

#### 3.1.9. Capnography

Literature regarding the use of capnography as a predictor of intubation in overdose is mixed. Millane et al. noted capnography was useful for providing real-time, reliable monitoring of ventilatory function and predicted 70% of hypoxic episodes in sedated nonintubated, poisoned patients [38]. However, in a prospective study in a French ED, with clinicians masked to end-tidal CO_2_ measurements, Viglino et al. demonstrated that capnometry in isolation could not adequately predict early complications in poisoned patients—with a sensitivity of 46%, and a positive predictive value of 33% [28].

#### 3.1.10. Bispectral Index (BIS) Monitoring

BIS monitoring involves signal processing of a patient's brain signals and is commonly used to assess the depth of anaesthesia. In a small cross-sectional study published in 2018, Eizadi-Mood et al. examined the use of BIS as a predictor for intubation in mixed drug poisoning in a small, unblinded observational study in Iran [34]. Fifty-eight patients were included, of whom 25 were intubated. BIS at admission was significantly higher for nonintubated patients (85.2 ± SE 1.7) compared with intubated patients (66.5 ± SE 2.6), a higher score indicating a higher conscious level. They concluded that a BIS of < 79.5 had a sensitivity of 88% and specificity of 87% for endotracheal intubation. Lee et al. examined 32 intoxicated patients with BIS in a prospective observational study in South Korea [22]. The researchers compared BIS to GCS and found BIS an effective index for predicting intubation (*p* 0.012), with a sensitivity of 91% and specificity of 50%.

#### 3.1.11. Acute Physiology and Chronic Health Evaluation (APACHE II)

Two studies examined the use of the APACHE II [10, 30]. APACHE II predicts ICU mortality, but it is calculated retrospectively and includes several biochemical values, and consequently is not readily available or calculable at the time of decision to intubate [51]. El-Sarnangawy and Hafez examined 104 overdose patients admitted to the Tanata Toxicology Unit in Egypt with a reduced level of consciousness, 24 of whom required intubation [30]. APACHE II scores > 18 demonstrated sensitivity and specificity of 67% and 90% for predicting the need for endotracheal intubation [30]. In predicting the need for intensive care interventions (including intubation), Hamad et al. examined 199 adult patients with overdose admitted to a medical ICU in a public medical centre in New York, USA [10]. Comparing intubated to nonintubated patients, the APACHE II score was significantly higher in the intubated group [10].

#### 3.1.12. Rapid Acute Physiology Score (RAPS) and Rapid Emergency Medicine Score (REMS)

El-Sarnagawy and Hafez examined the use of the RAPS and the REMS in the same patient cohort [30]. RAPS is an abbreviated version of APACHE II using parameters available on patients in the ED with scores from 0–16 [52]. RAPS was significantly higher in the 24 intubated patients, with a sensitivity of 50% and a specificity of 90%. REMS utilises similar observations to RAPS and includes age, with scores up to 26. REMS was higher in the intubated group and had a sensitivity of 68% and a specificity of 100% in predicting endotracheal intubation. The authors concluded that REMS had the highest positive predictive value (100%), identifying all cases that required mechanical ventilation.

Buswell et al. summarised the results of El-Sanagaway along with four other studies in a short cut review of the literature in 2019 [49]. None of the other included studies focused on a specific scoring system, but did report on the utilisation of GCS and other physiological parameters that were discussed previously. They noted that REMS requires prospective evaluation and reiterated the importance of clinical judgement.

#### 3.1.13. Cardiac Conduction, Oxygenation, Blood Pressure, Respiratory Rate, Awareness (COBRA)

**Figure 3:**
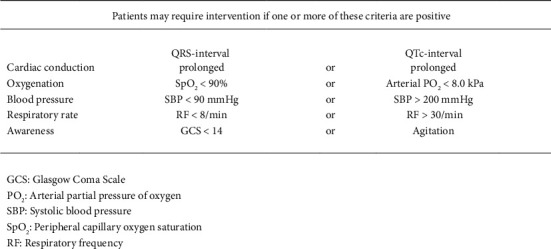
COBRA: decision rule for patients with intentional overdose of drugs having potential acute effects on neurological, circulatory or ventilatory function.

Wiersma et al. examined the use of the COBRA tool for predicting the need for ICU interventions (including intubation) in patients with overdose [44]. The COBRA tool consists of five parameters: cardiac conduction, oxygenation, blood pressure, respiratory rate and awareness (Figure 3). Of 230 included cases, 144 were admitted to the ICU; six required intubation. The negative predictive value of COBRA was 95.6% with a sensitivity of 100% and specificity of 61.1%. Note that this was used to predict any ICU intervention (40 patients) and was not focused on intubation alone.

#### 3.1.14. Tanta University Risk Model

The Tanta University Risk Model scores for complicated clinical courses in acutely poisoned patients. Of note, the type of drug is not required to make the calculation, the values are easily obtainable, and the variables are common in critical care settings, including GCS, oxygen saturations, diastolic blood pressure, respiratory rate and bicarbonate. Schmoll et al. examined its utility in a retrospective chart review of 293 poisoned patients, correlating a positive risk model score with ICU admission (intubation or vasopressors) [47].

#### 3.1.15. Outcomes for Observation vs. Intubation in Gamma-Hydroxybutyrate GHB Overdose

Munir et al. observed 170 patients with GHB overdose presenting to an Australian ED, 91 of whom had low GCS (< 8); 79 (86%) were not intubated [17]. There were no serious adverse outcomes [17]. Similar results were observed by van Helmond and Gresnigt and Horynaik et al. [26, 36]. van Helmond and Gresnigt found that conservative airway management for GHB patients with reduced GCS was safe [36]. For the 209 patients included, all were managed with the conservative strategy established at their facility in the Netherlands. Only 1.5% of patients (*n* = 3) underwent intubation, and all were discharged after a short stay in the ICU. Of note, 46 (22%) patients in this cohort did not maintain airway patency—with one receiving intubation, the rest being managed with basic airway manoeuvres and basic adjuncts. Similarly, 27 (12.9%) patients had an episode of hypoxia; all were managed successfully with simple oxygen supplementation. Thorn et al. demonstrated that intubated GHB patients were more likely to undergo CT brain imaging than conservatively managed patients [35].

#### 3.1.16. Length of Hospital Stay in GHB Overdose

Nonintubated patients with GHB overdose were discharged from the hospital sooner than those who were intubated [25, 26, 35]. Dietze et al. conducted a retrospective review of GHB presentations at two major inner-city EDs in Melbourne, Australia [25]. In one ED, patients with a GCS of < 8 were intubated; in the other, uncomplicated patients with a GCS < 8 were observed. Intubation, compared with conservative airway management, was associated with increased mean ED LoS (41 min (95% CI 19–65)) and increased odds of hospital admission of 9.9 (95% CI 2.4–41.9). Similarly, Thorn et al. completed a retrospective analysis of GHB presentations comparing intubated and conservatively managed patients [35]. Of 332 presentations, 280 were managed conservatively and 52 intubated. Median (IQR) total ED LoS was 24 h (4–123) for the intubated group and 5 h (0.5–84) for the conservatively managed group.

#### 3.1.17. Coingestants in GHB Overdoses

Several studies commented on polypharmacy with GHB-related presentations [11, 21]. Galacia et al. examined 505 patients presenting with GHB overdose, 385 (76%) of whom had taken other substances including alcohol, amphetamines, cocaine, ketamine and cannabis [21]. Patients with polypharmacy had a longer time to complete recovery of consciousness and a higher percentage needed mechanical ventilation compared with those who only ingested GHB. Couper et al. examined blood specimens from 146 GHB overdose patients, finding multiple other drugs including alcohol, marijuana, methamphetamine and cocaine [11].

## 4. Discussion

A review of the available literature identified several recurring themes influencing airway management decisions in overdose patients, including: age, sex, comorbidities, drug type, polypharmacy, aspiration risk, level of consciousness and the role of noninvasive monitoring. After data synthesis, four narrative themes were identified: (1) risk stratifying the decision to intubate; (2) monitoring for patients with overdose; (3) scoring systems and predicting the need to intubate; and (4) targeted GHB-specific literature.

### 4.1. Risk Stratifying the Decision to Intubate

Several studies focused on risk stratification of the decision to intubate and the associated need for ICU admission, based on patient factors and drug characteristics.

In terms of risk stratification, older age and male sex are overall associated with higher intubation rates for patients with reduced consciousness due to drug overdose [32, 33, 40, 43, 46]. It is difficult to draw causality from this; older male patients may be more likely to overdose drugs that are more likely to require intubation. It is likely that (in general) younger patients reflect those more likely to be affected due to recreational misadventure. Patients with comorbidities [2] and polypharmacy [9, 14, 27] are associated with a higher risk profile than patients with a single agent or alcohol only ingestion, which seem to be predictors of a benign outcome not requiring ICU [33].

The conflicting evidence on the use of the GCS in patients with drug overdose reflects the heterogeneity of the group and the low quality of available studies on the topic. Outcomes were difficult to compare as some studies examined GCS as related to ICU admission, others directly the need for intubation. Thus, there is no consensus as to reduced GCS as a mandate for intubation in overdose patients. Interestingly, this lack of consensus was reflected in a questionnaire put to Canadian emergency physicians by Munn et al. in 2020, with some mandating intubation for a GCS of less than eight, while others felt comfortable observing [39].

There may be value in using the GCS, in combination, with other clinical observations and an individualised risk assessment of the patient. In a ‘short cut' review of the literature, Buswell et al. summarised that although GCS may correlate with a need for intubation, in suitable patients, observation with airway adjuncts is appropriate [49]. There is heterogeneity of the patients and drugs involved in these cases. We suggest that select overdose patients can be safely observed despite a low GCS, and future research needs to evaluate this individualised approach [53]. It is likely that these patients are younger, with no comorbidities, have normal observations in the ED including oxygen saturations and respiratory rate and are more likely to have taken an accidental recreational overdose rather than a large intentional overdose. However, it should be reiterated that existing literature is of low-quality evidence, and that clinical judgement remains paramount, in combination with an individualised risk assessment for every patient.

### 4.2. Monitoring for Patients With Overdose

Overall, the evidence available on noninvasive monitoring to predict intubation is too limited to support or oppose the use of BIS or capnography [28, 34, 38]. However, they may be helpful in conjunction with other noninvasive observations to detect early clinical deterioration.

### 4.3. Scoring Systems

Similar to the theme of using clinical parameters to predict endotracheal intubation in order to identify at-risk patients early, several studies examined the use of clinical scoring systems in the prediction of ICU admission and endotracheal intubation.

Of the different scoring systems examining overdose patients, only the RAPS scoring system evaluation focused on the need for intubation and ventilation [30]. Others looked at composite predictors of ICU admission. Scoring systems using commonly available ED observations and values without requiring information on the specific drugs involved may be more useful. All concluded that scoring systems must be used in conjunction with clinical assessment. Given the heterogeneity of the patient group, it appears one can either aim for a very sensitive, or a very specific tool, but would be unable to achieve the sort of ‘gold standard' tool where both were high.

### 4.4. Targeted Literature: GHB Overdose

Overdose with GHB was the focus of several papers [11, 16, 17, 21, 25, 26, 35, 36]. GHB was initially developed in the mid-20^th^ century and subsequently found to occur endogenously. It is colourless and odourless. Used recreationally, it can cause euphoria and disinhibition, similar to the effects of 3,4-methylenedioxymethamphetamine (MDMA, also known as ecstasy) and alcohol. GHB has a narrow therapeutic index and a short plasma half-life of 30–60 min. Higher doses can lead to respiratory depression, bradycardia and reduced conscious level, with profound coma.

Historically, patients with GHB overdose were intubated for airway protection. However, its effects are short-lived with coma usually resolving within four to 6 hours. With more familiarity with GHB, there has been a trend towards observing, rather than intubating, these patients. Several observational studies examine intubation vs observation, length of hospital stay, the effect of coingestants and the length to time to normal GCS [11, 16, 17, 21, 25, 26, 35, 36].

GHB presentations are commonly managed conservatively. Although literature is observational, there does not appear to be an increase in adverse events compared with those who are intubated. Unsurprisingly, LoS is longer for those who are intubated, and polypharmacy likely impacts the duration of coma. Conservative airway management for GHB presentations can be safe, might shorten overall LoS and decrease some of the risks associated with intubation. Although these patients are often young without comorbidities and taking GHB recreationally, a key caveat is that the treating clinician needs to be confident that the patient does not have any other complicating factors, such as trauma or coingestants.

To date, there is only one RCT on the airway management of patients with reduced conscious level due to drug overdose [45]. Other studies are observational in nature, often with small sample sizes. The quality of the evidence is such that it is not clear what the ‘best' management option is for these patients. Many studies aimed to identify predictors of clinical outcome or comment on accurate monitoring to help clinical decision-making, but were limited by their study design and resultant bias.

## 5. Limitations

As this was a scoping review, no quality assessment or meta-analyses were conducted. The heterogeneity of patients, interventions and settings may limit generalisability. Only one RCT was included; other studies were observational.

## 6. Conclusion

The available literature on the approach to airway management in adult patients with overdose and decreased level of consciousness is heterogeneous and largely descriptive in nature. Individual clinical risk stratification is required for all patients, and triggers for intubation may need to be based on the clinical setting, patient profile and ingested agents. The findings from this scoping review can inform future research on this topic, such as a clinical decision tool for intubations in an ED setting. A prospective multicentre evaluation of such a tool may help articulate a more unified and evidence-based approach.

## Figures and Tables

**Table 1:** Characteristics of included studies.

Reference (primary author)	Publication year	Country	Study design	Centres	Department
Chan et al. [8]	1993	Australia	Prospective study	Single centre	Emergency department

Heyman et al. [9]	1996	USA	Retrospective chart review, convenience sampling	Single centre	Emergency department

Hamad et al. [10]	2000	USA	Chart review, retrospective	Single centre	Intensive care unit

Couper et al. [11]	2004	USA	Retrospective chart review	Single centre	Emergency department

Heard and Bebarta [12]	2004	USA	Prospective observational study	Single centre	Emergency department

Kelly et al. [13]	2004	UK	Prospective cohort study	Single centre	Toxicology unit

Testori et al. [14]	2006	Austria	Retrospective data analysis	Single centre	Emergency department

Adnet et al. [15]	2008	France	Prospective observational study	Single centre	Toxicology unit

Galicia et al. [16]	2008	Spain	Retrospective cohort study	Two hospitals	Emergency department

Munir et al. [17]	2008	Australia	Retrospective chart review	Single centre	Emergency department

Donald et al. [18]	2009	UK	Prospective parallel group comparison	Single centre	Emergency department

Duncan and Thakore [19]	2009	UK	Prospective observational study	Single centre	Emergency department

Kant et al. [20]	2010	Singapore	Retrospective observational study	Single centre	Toxicology unit

Galicia et al. [21]	2011	Spain	Observational study	Single centre	Emergency department

Lee et al. [22]	2011	South Korea	Retrospective study	Single centre	Emergency Department

Sabzghabaee et al. [23]	2011	Iran	Prospective follow-up study	Single centre	Emergency department

Eizadi-Mood et al. [24]	2011	Iran	Prospective data collection and retrospective analysis	Single centre	Emergency department

Dietze et al. [25]	2014	Australia	Retrospective paper and electronic audit	Two hospitals	Emergency department

Horyniak et al. [26]	2014	Australia	Prospective blinded study	Two hospitals	Emergency department

Maignan et al. [27]	2014	France	Retrospective observational study	Single centre	Intensive care unit

Viglino et al. [28]	2014	France	Prospective blinded study	Single centre	Emergency department

Beauchamp et al. [29]	2016	USA	Retrospective review	77 hospitals	Registry data

El-Sarnagawy and Hafez [30]	2016	Egypt	Prospective observational study	Single centre	Toxicology unit

Maarouf	2016	Tunisia	Retrospective study	Single centre	Emergency department

Van Den Berg et al. [31]	2016	The Netherlands	Retrospective cohort study	Two hospitals	Intensive care unit

van den Oever et al. [32]	2016	The Netherlands	Retrospective cohort study	Single centre	Intensive care unit

Viglino et al. [28]	2016	USA	Blinded prospective study	Single centre	Emergency department

Brandenburg et al. [33]	2017	The Netherlands	Retrospective cohort study	86 hospitals	Intensive care unit

Hua et al. [2]	2017	USA	Secondary data analysis from a prospective cohort	Two hospitals	Emergency department

Barbis	2018	Australia	Retrospective data extraction	Single centre	Emergency department

Eizadi-Mood et al. [34]	2018	Iran	Cross-sectional study	Single centre	Toxicology unit

Thorn et al. [35]	2018	Australia	Retrospective analysis	Single centre	Emergency department

van Helmond and Gresnigt [36]	2018	The Netherlands	Retrospective chart review	Single centre	Emergency department

Mehrl et al. [37]	2020	Germany	Retrospective chart review	Single centre	Emergency department

Millane et al. [38]	2020	Australia	Observational study—short report	Single centre	Emergency department

Munn et al. [39]	2020	Canada	Online questionnaire	N/A	N/A

Savage et al. [40]	2020	Australia	Retrospective data review	Single centre	Emergency department

Eizadi-Mood et al. [41]	2022	Iran	Cross-sectional study	Single centre	Toxicology unit

Kunzler et al. [42]	2022	USA	Airway registry study	25 hospitals	Emergency department

Miro	2022	Europe (24 countries)	Longitudinal analysis stratified by sex	36 hospitals	Emergency department
			Retrospective observational		

Nagashima et al. [43]	2022	Japan	Retrospective study	Single centre	Emergency department

Wiersma et al. [44]	2022	The Netherlands	Observational cohort study	Single centre	Emergency department
			Single centre, prospectively included, retrospective decision rule application		

Freund et al. [45]	2023	France	Randomised controlled trial	21 hospitals	Emergency department/intensive care unit

Noseda et al. [46]	2023	Europe (24 countries)	Retrospective multicentre matched case–control study	40 hospitals	Emergency department

Schmoll et al. [47]	2023	Germany	Retrospective chart review	Single centre	Intensive care unit

Abbreviations: UK = United Kingdom, USA = United States of America.

**Table 2:** Main outcomes of included studies.

Reference	Year	Intervention	Comparator	Outcomes	Conclusions
Chan et al. [8]	1993	Intubation	No intubation	The intubated group was older	GCS ≤ 8 was a useful indicator for intubation
				GCS ≤ 8, 37 intubated, 18 not intubated	Intubated group were older
				No respiratory complications in the nonintubated group	
		Ventilation		GCS ≤ 8, sensitivity 90%, specificity 95% intubation	Older patients with GCS ≤ 8: consider intubation
				Gag reflex assessed in 393. 41 intubated, 11 had a gag	

Heyman et al. [9]	1996	Intubation	No intubation	50 types of substances were isolated.	Intentional drug overdose accounted for 4.8% of ICU admissions.
				24 (56%) patients took more than one substance.	
				Antidepressant (30.2%).	
				Analgesia (23.2%).	
				Alcohol (18.6%).	
				2 patients died.	
				APACHE II 1–29 means score of 8.	
				GCS for 33: 13 had score of 15, 5 had score 8 or less.	Level of consciousness was best predictor of ICU admission and/or death.
				3 had a seizure	
				5 were intubated in the ED	
				15 ECG abnormality in ED	
				Charcoal in 76.7%, gastric lavage in 60.5%	ECG was not a good predictor.
				LoS 1–21 mean 2	
				41 (95%) survived to discharge	

Hamad et al. [10]	2000	Morbidity and mortality including intubation	No morbidity /mortality	2.7% mortality.	Divided into mortality and nonmortality for all patients admitted to ICU.
				35% morbidity: intubation, pneumonia, arrhythmia, ECG changes, hypotension.	
				16% had more than one morbidity.	Age, GCS and APACHE II were all significantly different.
				Most intubations in ED.	GCS and APACHE II significant comparing those requiring/not requiring mechanical ventilation.
				4.5 hr average in ED.	Majority of OD patients admitted to ICU did not develop major morbidity or mortality.
				Morbidity vs nonmorbidity: age, GCS (8.2 vs. 13.1) and APACHE II scores significant difference.	Age significant factor—those > 65 much more likely to require intubation than those < 65.
				GCS lower in intubated vs nonintubated, APACHE II higher.	APACHE II and GCS are useful.
				$1575/night costs.	RR may be helpful.

Couper et al. [11]	2004	Intubation	No intubation	GHB was detected in 37% of patients with presumed GHB intoxication based on clinical features.	Patients who overdosed on GHB presented with a markedly decreased level of consciousness.
				Alcohol was measured in 41% of GHB +ve patients and in 48% of GHB −ve patients.	
				40% of these patients fell unconscious or were found unresponsive at a club, dance club or rave.	Coingestion of ethanol or other drugs was common, as were bradycardia, hypothermia, respiratory acidosis and emesis.
				2/3rds were unconscious or unresponsive on admission.	
				Other common symptoms were drowsiness, agitation, ataxia, vomiting, sweating, lack of gag reflex, respiratory depression.	Typically, the patients regained consciousness spontaneously within 5 hr of the ingestion.
				34 pts required intubation, average LoS in ED was 1.5–6 hrs	

Heard and Bebarta [12]	2004	GCS evaluation by doctors	GCS evaluation by nurses	44 evaluations by doctors, 16 by nurses and 18 not identified.	GCS is a reliable tool for reporting mental status of poisoned patients in the ED and can be calculated by doctors or nurses.
				16/39 had GCS 15.	
				Agreement between doctor and nurse was good, good inter-rater reliability.	A variety of common poisonings were observed.

Kelly et al. [13]	2004	AVPU	GCS	A approx. GCS 15.	AVPU responsiveness scale score corresponds to certain GCS as in primary results.
				V approx. GCS 12.	
				P approx. GCS 8.	
				U approx. GCS 3.	
				(In poisoned patients)	

Testori	2006	ICU admission and intubation	No intubation	29.3% could be discharged after outpatient treatment.	Mixed intoxications can cause serious complications.
				62.7% were admitted to the ED for further care.	
				6.4% required ventilation.	The group with the highest complication rate was those poisoned with narcotics.
				31.1% were admitted to a ward and 3.3% were transferred to ICU.	

Adnet et al. [15]	2008	GCS score at time of intervention	N/A	64% were intubated successfully at the 1st attempt.	Poisoned patients with 7 ≤ GCS ≤ 9 are associated with a high rate of difficult emergency intubations.
				In 71% of difficult intubation patients, glottic exposure was poor.	
				Median GCS was 6. 7 ≤ GCS ≤ 9 was associated with the highest incidence of intubation difficulty.	This is estimated to be due to the lack of deep coma coupled with less frequent use of sedation or RSI in this patient group.

Galicia et al. [16]	2008	Intubation	No intubation	89% of cases were treated between 0:00 and 08:00 and on weekends (89%).	Given the short duration of the coma and the usual absence of complications and mortality, the treatment of these patients with an overdose of GHB is general support.
				97% of patients came from public places.	
				72% had a decreased GCS, 49% of which had GCS < 8.	
				79% had other neuro symptoms, 19% GIT symptoms.	
				14% had bradycardia, mydriasis 63%, miosis 16%.	
				Most common coingestants were ethanol (53%) and cocaine (16%).	
				99% of patients were discharged within 12 hrs.	

Munir et al. [17]	2008	Intubation	No intubation	74% of attendances were on Saturday or Sunday, × 26 more frequent on public holidays.	Altered LOC primary presenting symptom.
				36% were GHB ingestion alone, most common coingestant was ecstasy.	
				Altered LOC was the primary presenting symptom, 51% had airway interventions; OPA 59%, NPA 26%, 8% ETI.	
				87% of patients with low GCS were not intubated.	
				39% were bradypnoeic at ED.	Despite 54% presenting with low GCS, only 8% were intubated or transferred to ICU (2%).
				Hypothermia occurred in 70%.	
				75% had respiratory acidosis, 16.5% vomited.	

Donald et al. [18]	2009	Intubation	No intubation	4 patients with GCS 3 were Mx with OPA.	With a combination of thorough clinical and physiological assessment and close observation in the correct environment, a select group of poisoned patients can be managed safely without the need for intubation and ICU admission.
				RSI was performed in ED on 11/12 intubated, one failed and required LMA rescue device.	
				Reasons for intubations were loss of airway protection (9), failure of ventilation/oxygenation in (6) and predicted clinical course (8).	
				4 intubated group had head CTs, all were normal.	
				None of the nonintubated group was scanned.	
				Mean LOS for nonintubated patients was 26 hrs.	
				Mean ICU LOS for intubated patients was 1.7 days and mean total hospital stay was 5.4 days.	

Duncan and Thakore [19]	2009	Intubation	No intubation	Median GCS on admission to SS was 11.	It can be safe to monitor and observe patients with reduced LOC in a short-stay ward.
				12 patients had GCS ≤ 8 on admission, none had clinically significant aspiration or required intubation, and all had GCS 15 on discharge.	The overall incidence rate for intubation was 1.4% and aspiration 0%.
				Median LOS was 26 h for those GCS ≤ 8 and 14 h for those GCS > 8.	
				65% of short stay poisoning admissions were intentional overdoses.	Isolated alcohol intoxication had the lowest median GCS but had the most rapid recovery.
				39% of patients consumed alcohol.	
				14 patients required airway support on admission.	33% of those with a GCS ≤ 8 were not intubated and none developed respiratory complications.
				1 patient required intubation and ICU admission.	

Kant et al. [20]	2010	Intubation	No intubation	8.5% required HDU/ICU.	Psychiatric medications and benzodiazepines were the commonest class of agents associated with HDU or ICU admissions, while cardiotoxic drugs were the most lethal.
				Of those admitted to HD or ICU, 60% were male, and the commonest age group was 31–40 years old.	
				46% of these patients required intubation.	
				Deliberate self-poisoning or drug abuse accounted for 70% of cases.	
				Median HDU and ICU LOS were 37.5 hrs and 41.8 hrs.	

Galicia et al. [21]	2011	Intubation	No intubation	24% GHB intoxication alone, 76% coingestants.	Admissions due to GHB were more frequent during the weekend and at night.
				The motive for seeking medical attention was reduced LOC in all cases.	
				84% had GCS < 15 at ED admission.	Reduce LOC was the main reason for ED admission, 91% were D/C < 12 hr.
				Severely reduced LOC was more frequent in the group that claimed coingestants.	
				Most frequent symptoms: neurological, behavioural, GIT, resp and cardiovascular.	Antidotes seemed to have no impact on poisoning unless there was simultaneous benzodiazepine or opiate ingestion.
				Mechanical ventilation as required for 3%.	

Lee et al. [22]	2011	BIS	GCS	32 of 126 intoxication patients were enrolled.	In sedative-intoxicated patients, BIS is useful in recognising the degree of sedation, predicting consciousness recovery time and as an objective index of intubation.
				Mean age was 50.94 ± 18.01. BIS was 39–88.	
				BIS was an effective index for intubation (*p*=0.012) showing sensitivity of 91% and specificity of 50%.	
				When set to 77.5, sensitivity and specificity were 59% and 100%, respectively.	

Sabzghabaee et al. [23]	2011	Complications, including intubation	Without complications	20 had complications.	GCS ≤ 10 higher chance of complications.
				12 had severe complications.	Significant difference in the mean value of each component of the GCS as well as the overall GCS between patients with/without complications.
				2 died.	Logistic regression that chance of complication is 44 times higher for patients with motor score less than 5, similar (higher) for eye and verbal.

Eizadi Mood et al. [24]	2011	Survivors	Nonsurvivors	Significant differences in APACHE II, GCS but only GCS had difference at time 0 and time 24—e.g., GCS a good predictor.	GCS, APACHE II at 24 hr and APACHE II at 0 hr seem to predict the outcomes in mixed drug poisoning patients more accurately.
				Discrimination was excellent for GCS at 24 hrs, APACHE II 24 hours and APACHE II 0 hrs, e.g., these scoring systems seemed to predict the outcomes in mixed drug poisoning.	

Dietze et al. [25]	2014	Intubation	No intubation	Most presentations occurred early in the morning on weekends.	Intubation was associated w/average increase ED LoS of 41%.
				33% had cointoxicant drugs, 33% had coingestion of alcohol, 59% presented w/ GCS ≤ 8.	
				Average ED LOS was 3.6 hrs, 7% resulted in admission. 16% of patients presented to ED intubated, 84% were extubated in the ED.	

Horyniak et al. [26]	2014	Intubation	No intubation	69% of patients were BIBA, 42% from public places, 26% from private residences.	Although the majority of presentations were effectively treated, with discharge within a short time frame, the number and timing of presentations places a significant burden on EDs.
				< 10% reported their last location as a dance party or music festival.	
				80% had no history of physical or psychiatric illnesses.	
				The most common substance was GHB (36%), and 89% of GHB presentations were related to altered LOC with 55% having GCS ≤ 8.	
				17% of GHB patients were intubated.	
				Treatment was mainly patient observation, with intervention limited to IVF and paracetamol in most cases.	
				14% of all pts were admitted to hospital, mainly to ED SSU (65%). 81% of patients were able to be discharged home directly.	

Maignan et al. [27]	2014	ICU admission and intubation	N/A	142 admitted to ICU.	GCS was relevant, delay to ED, drug dose and some drug categories also relevant.
				GCS significantly lower in ICU admitted patients.	
				98 were intubated (all in ICU group).	
				Greater uncertainty about drugs involved in the ICU group.	BP, HR and sats were not linked to ICU admission.
				ICU patients ingested more.	
				Cardiac drug, neuroleptic, meprobamate, ingestion to ED evaluation interval, number of tablets, presumed toxic dose, GCS > all significant variables.	

Viglino et al. [28]	2014	Complications'	No complications	15/104 exhibited at least 1 complication, ETCO_2_ ≥ 50 predicted the occurrence of a complication with 46.6% sensitivity and 75.9% specificity.	Preliminarily, the monitoring of ETCO_2_ cannot adequately predict early complications in self-poisoned patients.
				GCS on admission was as powerful as ETCO_2_ to detect complications.	

Beauchamp et al. [29]	2016	Intubation	N/A	Most common single substance exposures managed with intubation were sedative hypnotics (9.8%), antidepressants (8.7%) and opiates (8%).	Knowledge of substances commonly involved in exposures mx with intubation may inform triage and resource planning in the ED resuscitation of critically ill poisoned patients.
				Polysubstance exposure occurred in 29%.	
				Decontamination + elimination was used in 12.8%.	
				The most common clinical features were CNS depression (49.5%) and respiratory depression (39.1%).	

El-Sarnagawy and Hafez [30]	2016	Intubation	No intubation	24/104 patients required intubation. GCS ≤ 8 had 100% sensitivity for mechanical ventilation and 100% NPV.	Admission REMS is a valuable prognostic tool for mechanical ventilation.
				Patients who were intubated had significantly higher APACHE II on admission and recommended cut-off value > 17 (86% sensitivity and 73% specificity).	
				RAPS was significantly higher in mechanically ventilated patients (sensitivity 50% and specificity 90%).	The combined use of GCS + REMS is recommended as GCS > 8 had NPV 100% and REMS > 8 had PPV 100%.
				REMS in mechanically ventilated patients was significantly higher, 100% specificity at cut-off > 8 and PPV 100% and NPV 90.9%	

Maarouf	2016	ICU admission and intubation	N/A	Toxic ingestion was voluntary in 87%.	A history of previous ICU admission for poisoning, GCS < 13 and an anticholinergic toxidrome are prognostic factors for ICU admission.
				23% had psychiatric past history.	
				Initial exam findings were decreased GCS w/coma, digestive disorders, hypotension and fasciculations.	
				Toxidromes were identified in 55%. 59% of cases were admitted to the ICU.	

Van Den Berg et al. [31]	2016	Intubation	No intubation	5.8% of patients not ventilated on ICU admission develop the need for intubation and mechanical ventilation in the next 24 hrs.	GCS on admission is a significant predictor for the need of mechanical ventilation.
		Ventilation		25% of patients admitted with GCS < 8 were intubated and ventilated in the first 24 hrs, whereas only 2.6% of patients admitted with a GCS ≥ 8 needed tracheal intubation and ventilation in the first 24 hrs after ICU admission.	

van den Oever et al. [32]	2016	Emergency treatment	No emergency treatment	41% of patients received some form of emergency treatment.	ICU interventions could have been reliably predicted by clinical assessment, supplemented with ECG and blood gas analysis, in the ED.
				Median time spent in HCU/ICU was 18.02 hr.	
				77% were defined as high acuity (had ≥ 1 of the 6 defined predictors).	
				Low acuity patients were significantly younger and more likely to be treated w/ activated charcoal or intestinal lavage.	
				Only high acuity patients received antidotes in ED.	

Viglino et al. [28]	2016	Predictive value of ETCO_2_	N/A	ETCO_2_ ≥ 50 mmHg at any time during monitoring had a sensitivity of 46% and a specificity of 80% for detecting early complications.	Capnometry in isolation does not provide adequate prediction of early complications in self-poisoned patients referred to the ED.
				PPV 33% and a NPV of 88%.	

Brandenburg et al. [33]	2017	Mechanical ventilation	N/A	6.5% of patients admitted to ICU required ICU-level management.	If this prediction model were used in clinical practice, the observational admissions of intoxicated patients would be reduced by 34.3%.
				The strongest predictors for ICU management were resp insufficiency, age > 55 and GCS < 6.	
				Predictors that ICU management was likely unnecessary were alcohol and other poisonings and SBP ≥ 130.	
				The prediction model had 93.4% sensitivity and 98.7% NPV.	
				5 most common diagnoses in combination with intoxication were coma/change in LOC (10.2%), medical resp problems (5.5%), aspiration pneumonia (5.3%), endocrine/metabolic disturbances (4.8%) or drug toxicity (4.5%).	

Hua et al. [2]	2017	Intubation	No intubation	Incidence of ETI 3.5%, risk factors for ETI included younger age.	Younger age was associated with ETI.
				Most common indication for ETI was perceived worsening of clinical course.	The early complication rate for ETI was very low overall.

Barbis	2018	Intubation	No intubation	Exposure, physical parameters, GCS, intubation status, presence of aspiration pneumonia, LoS and mortality	Intubated group had a higher min recorded O_2_ sats (100% vs. 97%), and lower admission presenting HR (mean 90 vs. 100 bpm). tGCS was better than mGCS at predicting intubation status. Sensitivity was 76% and 89% for mGCS and tGCS, respectively (specificity 86% and 82%). Cut-point to predict intubation: tGCS = 8.5, mGCS = 4.5. 15 cases with a GCS < 9

Eizadi-Mood et al. [34]	2018	Intubation	No intubation	43.1% of patients required intubation.	BIS is an appropriate index for the prediction of the need to intubation in poisoned patients with ingestion of different drugs.
				At the admission time, the mean SE BIS index value for poisoned patients who needed endotracheal intubation was 66.47 ± 2.57 in comparison with 85.21 ± 1.47 for patients who did not need intubation.	

Thorn et al. [35]	2018	Intubation	No intubation	GCS < 9 documented in 78 nonintubated and 51 intubated patients.	GCS was the only significantly different clinical parameter on presentation between intubated and nonintubated patients.
				Median lowest GCS for nonintubated was 7, intubated 3.	
				Median time to extubation was 7 hr vs. nonintubated patient time to GCS 15 2.5 hr.	
				Median total LoS: intubated 24 h vs nonintubated 5 hr.	

van Helmond and Gresnigt [36]	2018	Intubation	No intubation	> 75% had GCS < 9, median GCS 6. 1.9% ETT, 206/209 were observed in the ED while monitoring rhythm, PR, RR, O_2_ sats and capnography.	Supportive care, monitoring of vital signs and neurologic state, and noninvasive airway management were sufficient to prevent major adverse events.
				Mean ED LoS 156 min.	

Mehrl et al. [37]	2020	Intubation	No intubation	53 had GCS ≤ 8.	The majority of comatose acutely intoxicated patients could be managed safely without ETI.
				8/53 were intubated.	
				Aspiration pneumonitis was diagnosed in 2/45 nonintubated patients and 2/8 intubated patients.	

Millane et al. [38]	2020	ETCO_2_ in nonintubated patients	N/A	Capnography provided a continuous trace for 99% of monitored study time.	Capnography's ability to provide a continuous reliable measurement of ETCO_2_ and to signal measurement interruption has the potential to reduce patient–nurse ratios, while maintaining safe monitoring.
				2 patients did not maintain a continuous ETCO_2_.	
				Capnography detected 15 episodes of apnoea, 10 episodes minor hypoxia.	

Munn et al. [39]	2020	Intubation	No intubation	17.4% considered GCS < 8 an independent indicator for intubation, 17% agreed intubation was the standard of care for ethanol intoxication.	It is acceptable to avoid intubation in a subset of intoxicated and postseizure ED patients. Most ED physicians do not consider GCS ≤ 8 as an absolute indication for intubation.

Savage et al. [40]	2020	ICU admission and intubation	N/A	Median age of pts requiring ICU admission vs those not was 41 vs. 36.	A history of overdose, use of atypical antipsychotics and older age were risk factors for ICU admission.
				72.9% had a preexisting psychiatric diagnosis, depression being the most common (68.3%).	
				Personality disorders were more common in those admitted to ICU than those who were not (32.1% vs. 21.7%).	
				57.8% were polypharmacy OD.	
				Atypical antipsychotics were more commonly implicated in ODs requiring ICU admission than those who did not (30.7% vs. 16.2%).	
				ETT was the most common indication for ICU admission.	
				22.5% represented within 1 yr of their index OD and 30% represented within 4 yr.	
				Median time from the index presentation to next OD presentation was 133 d.	

Eizadi-Mood et al. [41]	2022	Interventions	N/A	In the minor group, opioids/opiates, alcohols and benzodiazepines (14.7%) were the most prevalent poisoning, multidrug (23.3%) was in the moderate and severe groups, and finally, pesticides' poisoning (23%) was most common in the fatal group.	Severity of poisoning increased with age.
					Most patients were male.
				The predictive factors for poisoning severity were prehospital antidote administration [OR (95% CI); *p* value) [7.08 (1.77–28.34); 0.006]; loss of consciousness [4.38 (1.84–10.42), 0.001]; abnormal ECG [4.56 (1.65–12.56); 0.003]; and time interval of poisoning to admission in the hospital [1.15 (1.02–1.28); 0.01).	Men more likely to have fatal poisoning.
					Majority of fatal poisonings related to suicide attempts.
				Patients without complications were observed in 49.7% of subjects.	Patients with severe and fatal poisoning had more interventions.
					Severe to fatal poisonings more likely to involve multidrug, pesticides and opiates.
				Patients with the loss of consciousness [66.06 (2.41–180.07); 0.01); underlying disease [3.65 (1.09–12.24); 0.03]; abnormal respiration [1.14 (1.02–1.27); 0.02); have had a greater risk of complications and death.	Patients with abnormal ECG had greater odds of severe poisoning.
					LoC was important in predicting severity and outcome.

Kunzler et al. [42]	2022	Intubation	N/A	11% intubated for overdose.	Intubation is less risky for OD compared with other indications.
				89% intubated for other indications.	OD patients may be safer to intubate.
				First attempt success 90.5% in OD patients, 87.5% in other patients.	OD patients had less complications.
				OD less hypotension, fewer adverse events, no surgical airways.	
				OD patients younger, less obese, less difficult airway.	Knowing the risk of intubation can inform the decision-making of clinicians at the bedside.
				OD patients more likely preoxygenation, apnoeic oxygenation, bougie use.	

Miro	2022	ICU admission and intubation	No ICU admission or intubation	10,344 hospitalised.	Males significantly more likely to be intubated.
				2568 ICU.	
					Hospitalisation, ICU and death similar by sex.
				1391 intubated.	1/4 hospitalised.
				171 died.	1/20 ICU.
					Severity in presentations remained unchanged over 6-year period.

Nagashima et al. [43]	2022	Intubation	No intubation	20 (15.2%) intubated.	Relating to drug overdose due to self-harm:
				Age 8 yr higher in the intubated group.	
				Antipsychotics, anticonvulsants and antidepressants had higher incidence of intubation.	- GCS at transport predicted intubation
				HR and RR higher in intubation, PO_2_ higher in intubation.	- Anticonvulsants/alert levels of same are associated with intubation (OR 27.8× higher when at alert level in blood)
				GCS effectively predicted intubation in drug overdose.	- Antipsychotics were associated with tracheal intubation (OR 2.5)

Wiersma et al. [44]	2022	ICU admission and intubation	N/A	NPV of COBRA was 95.6%, then at 3 hr was 100%.	NPV good.
				Sensitivity 100%.	Fluid resus common.
				Specificity 61.1%.	40 of 40 interventions were identified by the test.
				PPV 35.1%.	

Freund et al. [45]	2023	Conservative strategy of withholding intubation	Standard care	No patients died in either group.	A conservative strategy of withholding intubation had improved hierarchical composite end point outcome compared with traditional strategy.
				Intervention strategy of withholding intubation resulted in lower length of ICU stay and lower length of hospital stay.	
				Lower median length of ICU stay (0 hours [IQR, 0–18.5] vs. 24.0 hrs [IQR, 0–57.0]; rate ratio [RR], 0.39 [95% CI, 0.24–0.66]).	Half of patients were intubated in the control group, showing continued clinical equipoise.
				In the intervention group, the median length of hospital stay was 21.5 hrs (IQR, 10.5–44.5) compared with 37.0 hrs (IQR, 16.0–79.0) in the control group (RR, 0.74 [95% CI, 0.53–1.03]).	
				The hierarchical composite primary end point was improved in the intervention compared with the control group, with a win ratio of 1.85 (95% CI, 1.33–2.58; *p* < 0.001).	

Noseda et al. [46]	2023	Need for critical care	N/A	Age > 35, polydrug use, ethanol coingestion, use of GHB increased the odds of admission to critical care.	6% required crit care admission.
				Use of cocaine, cannabis, heroin, amphetamines and night-time ED presentation were less frequently associated with admission to critical care.	Male sex not associated with crit care admission.
					Arriving at night resulted in less crit care admission—unclear why.

Schmoll et al. [47]	2023	ICU admission and intubation	No ICU admission or intubation	Tanata University risk model score being positive is related to complicated clinical course in acutely poisoned patients.	The type of drug is not required to make the calculation.
				May reflect general physiologic markers indicating ICU requirement.	

Abbreviations: 95% CI = 95% confidence interval, APACHE II = acute physiology and chronic health evaluation II, AVPU = alert, verbal, pain, unresponsive, BIBA = brought in by ambulance, BIS = bispectral index, CNS = central nervous system, COBRA = cardiac conduction, oxygenation, blood pressure, respiratory rate, awareness, CT = computed tomography, ECG = electrocardiogram, ED = emergency department, ETCO_2_ = end-tidal carbon dioxide, ETI = endotracheal intubation, GCS = Glasgow Coma Scale, GHB = gamma-hydroxybutyrate, GIT = gastrointestinal, HDU = high dependency unit, HR = heart rate, ICU = intensive care unit, IVF = intravenous fluids, LMA = laryngeal mask airway, LOC = level of consciousness, LoS = length of stay, NPA = nasopharyngeal airway, NPV = negative predictive value, OD = overdose, OPA = oropharyngeal airway, OR = odds ratio, PPV = positive predictive value, RAPS = Rapid Acute Physiology Score, REMS = Rapid Emergency Medicine Score, RR = respiratory rate, RSI = rapid sequence intubation, SSU = short stay unit.

## Data Availability

Data and materials supporting results will be made available upon reasonable request by the corresponding author (Richard A. F. Pellatt) after consideration of the ethical implications of sharing the data.

## Nomenclature

BIS: Bispectral index
CT: Computed tomography
ED: Emergency department
ETI: Endotracheal intubation
GCS: Glasgow Coma Scale
GHB: Gamma-hydroxybutyrate
ICU: Intensive care unit
LOC: Level of consciousness
LoS: Length of stay
MDMA: 3,4-Methylenedioxymethamphetamine
OSF: Open Science Framework
PRISMA-ScR: Preferred Reporting Items for Systematic Reviews and Meta-Analyses Extension for Scoping Reviews
RCT: Randomised controlled trial
SE: Standard error
SRA: Systematic review accelerator
UK: United Kingdom
USA: United States of America

## Supporting Information

Supporting Information 1 Appendix 1: Search strategy.

Supporting Information 2 Appendix 2: Excluded studies.
