# Supplementary material for: Predictors and Outcomes of Airway Management in Patients Presenting to the Emergency Department With Overdose and Decreased Consciousness: A Scoping Review
Source: Emerg Med Int. 2025 Oct 4;2025:8071582. doi: 10.1155/emmi/8071582 (PMC12515096; doi:10.1155/emmi/8071582)
Supplement: Supporting Information 1 — Appendix 1: Search strategy. [file 8071582.f1.docx]

### Appendix I. Search strategy.

**PubMed (via NLM)**

("Drug overdose"[Mesh] OR Poisoning[Mesh] OR "substance-related disorders"[Mesh] OR "Sodium Oxybate"[Mesh] OR Overdose[tiab] OR Toxicology[tiab] OR Sedation[tiab] OR Poisoning[tiab] OR GHB[tiab] OR gamma-hydroxybutyrate[tiab] OR gamma-butyrolactone[tiab]) AND

("Emergency Service, Hospital"[Mesh] OR "Emergency Medicine"[Mesh] OR "Poison Control Centers"[Mesh] OR "Triage"[Mesh] OR Clinicians[tiab] OR "Emergency medicine"[tiab] OR "tertiary-care hospitals"[tiab] OR "University hospital"[tiab] OR "Emergency department"[tiab] OR "Emergency departments"[tiab] OR "Emergency ward"[tiab] OR "Emergency wards"[tiab] OR ED[tiab])

AND

("Airway Management"[Mesh] OR Capnography[Mesh] OR "Monitoring, Physiologic"[Mesh] OR "airway management"[tiab] OR "rapid sequence induction"[tiab] OR Intubation[tiab] OR Intubate[tiab] OR Intubated[tiab] OR "Observation unit"[tiab])

AND

("Respiratory Insufficiency"[Mesh] OR "Glasgow Coma Scale"[Mesh] OR Coma[Mesh] OR "Self-Injurious Behavior"[Mesh] OR "Coma scale"[tiab] OR "Organ dysfunction"[tiab] OR "Cardiovascular toxicity"[tiab] OR "Hemodynamic instability"[tiab] OR "Airway obstruction"[tiab] OR Aspiration[tiab] OR "Ventilatory monitoring"[tiab] OR "Ventilatory function"[tiab] OR Deaths[tiab] OR Admission[tiab] OR "Self-harm"[tiab] OR "Self harm"[tiab] OR "Ambulance transport"[tiab] OR Resuscitation[tiab] OR Mortality[tiab])

**Cochrane CENTRAL (via Wiley)**

([mh "Drug overdose"] OR [mh Poisoning] OR [mh "substance-related disorders"] OR [mh "Sodium Oxybate"] OR Overdose:ti,ab OR Toxicology:ti,ab OR Sedation:ti,ab OR Poisoning:ti,ab OR GHB:ti,ab OR gamma-hydroxybutyrate:ti,ab OR gamma-butyrolactone:ti,ab) AND

([mh "Emergency Service, Hospital"] OR [mh "Emergency Medicine"] OR [mh "Poison Control Centers"] OR [mh Triage] OR Clinicians:ti,ab OR "Emergency medicine":ti,ab OR "tertiary-care hospitals":ti,ab OR "University hospital":ti,ab OR "Emergency department":ti,ab OR "Emergency departments":ti,ab OR "Emergency ward":ti,ab OR "Emergency wards":ti,ab OR ED:ti,ab)

AND

([mh "Airway Management"] OR [mh Capnography] OR [mh "Monitoring, Physiologic"] OR "airway management":ti,ab OR "rapid sequence induction":ti,ab OR Intubation:ti,ab OR Intubate:ti,ab OR Intubated:ti,ab OR "Observation unit":ti,ab)

AND

([mh "Respiratory Insufficiency"] OR [mh "Glasgow Coma Scale"] OR [mh Coma] OR [mh "Self-Injurious Behavior"] OR "Coma scale":ti,ab OR "Organ dysfunction":ti,ab OR "Cardiovascular toxicity":ti,ab OR "Hemodynamic instability":ti,ab OR "Airway obstruction":ti,ab OR Aspiration:ti,ab OR "Ventilatory monitoring":ti,ab OR "Ventilatory function":ti,ab OR Deaths:ti,ab OR Admission:ti,ab OR Self-harm:ti,ab OR "Self harm":ti,ab OR "Ambulance transport":ti,ab OR Resuscitation:ti,ab OR Mortality:ti,ab)

**Embase (via Elsevier)**

("Drug overdose"/exp/mj OR intoxication/exp/mj OR "oxybate sodium"/exp/mj OR Overdose:ti,ab OR Toxicology:ti,ab OR Sedation:ti,ab OR Poisoning:ti,ab OR GHB:ti,ab OR gamma-hydroxybutyrate:ti,ab OR gamma-butyrolactone:ti,ab)

AND

("hospital emergency service"/exp/mj OR "Emergency Medicine"/exp/mj OR "poison center"/exp/mj OR Clinicians:ti,ab OR "Emergency medicine":ti,ab OR "tertiary-care hospitals":ti,ab OR "University hospital":ti,ab OR "Emergency department":ti,ab OR "Emergency departments":ti,ab OR "Emergency ward":ti,ab OR "Emergency wards":ti,ab OR ED:ti,ab)

AND

("respiration control"/exp/mj OR capnometry/exp/mj OR "airway management":ti,ab OR "rapid sequence induction":ti,ab OR Intubation:ti,ab OR Intubate:ti,ab OR Intubated:ti,ab OR "Observation unit":ti,ab)

AND

("respiratory failure"/exp/mj OR "Glasgow Coma Scale"/exp OR Coma/exp/mj OR "Coma scale":ti,ab OR "Organ dysfunction":ti,ab OR "Cardiovascular toxicity":ti,ab OR "Hemodynamic instability":ti,ab OR "Airway obstruction":ti,ab OR Aspiration:ti,ab OR "Ventilatory monitoring":ti,ab OR "Ventilatory function":ti,ab OR Deaths:ti,ab OR Admission:ti,ab OR Self-harm:ti,ab OR "Self harm":ti,ab OR "Ambulance transport":ti,ab OR Resuscitation:ti,ab OR Mortality:ti,ab)
