# Supplementary material for: Predictors and Outcomes of Airway Management in Patients Presenting to the Emergency Department With Overdose and Decreased Consciousness: A Scoping Review
Source: Emerg Med Int. 2025 Oct 4;2025:8071582. doi: 10.1155/emmi/8071582 (PMC12515096; doi:10.1155/emmi/8071582)
Supplement: Supporting Information 2 — Appendix 2: Excluded studies. [file 8071582.f2.docx]

### Appendix 2. Excluded studies.

1. Alghafees M, et al. Poisoning-related emergency department visits: the experience of a Saudi high-volume toxicology center. **Wrong study type. Not related to airway management.**

2. Aloise, M, et al. Accidental hashish ingestion in children: A Pavia Poison Centre case series. **Wrong population. Paediatrics.**

3. Arens A, et al. A 24-hour observation time is hard to swallow: Evidence for 8 hours of observation of methamphetamine stuffers. **Wrong study type. Focused on single drug.**

4. Aydogan, S, et al. The assessment of the risk of unplanned extubation in an adult intensive care unit. **Wrong setting. Intensive care.**

5. Bateman RM, et al. 36th International Symposium on Intensive Care and Emergency Medicine : Brussels, Belgium. 15-18 March 2016. Wrong study type.

6. Beauchamp GA, et al. Poisonings Associated with Intubation: US National Poison Data System Exposures 2000-2013. **Wrong outcome.**

7. Beauchamp GA, et al. Toxicological Emergencies in the Resuscitation Area of a Pediatric Emergency Department: A 12-Month Review. **Wrong population. Paediatrics.**

8. Binner C, et al. Munich Oktoberfest experience: remarkable impact of sex and age in ethanol intoxication. **Wrong study type. Focused on single drug.**

9. Bremen K, et al. Patients requiring ICU treatment for acute poisoning-a 20-year single-center retrospective : Acute poisoning in intensive care. **Wrong outcome. Focused on ICU setting.**

10. Burket, GA, et al. Endotracheal Intubation in the Pharmaceutical-Poisoned Patient: a Narrative Review of the Literature. **Wrong study type. Review article.**

11. Calello DP, et al. Observation unit experience for pediatric poison exposures. **Wrong population. Paediatrics.**

12. De Groot RI, et al. Risk factors for unplanned extubation in critically ill patients. **Wrong outcome. Focused on unplanned extubation.**

13. Descamps AMK, et al. Adults admitted to the emergency department of a university hospital in Belgium for acute poisoning with ethanol as a co-ingestant: Characteristics and direct medical costs**. Wrong outcome. Focused on cost.**

14. Driedger GE, et al. What are kids getting into these days? A retrospective chart review of substance use presentations to a Canadian pediatric emergency department. **Wrong population. Paediatrics.**

15. El-Gharbawy D, et al. A nomogram proposal for early prediction of intensive care unit admission in patients with acute antipsychotic poisoning. **Wrong exposure. Focused on single drug.**

16. Emerman CL, et al. Level of consciousness as a predictor of complications following tricyclic overdose. **Wrong study type. Focused on single drug.**

17. Eyer F, et al. Drug overdoses. **Wrong study type. Review article.**

18. Fischer, D, et al. Evaluation of the level of sedation and monitoring of intubated patients in the emergency department. **Wrong population.**

19. Fox L, et al. Clinical risk factors in Emergency Department (ED) patients with prescription opioid overdose. **Wrong study type. Focused on single drug.**

20. Fox L, et al. Clinical risk factors in ED patients with prescription opioid overdose. **Wrong study type. Focused on single drug.**

21. Fox LM, et al. Risk factors for severe respiratory depression from prescription opioid overdose**. Wrong study type. Focus on single drug.**

22. Hassanian-Moghaddam H, et al. Risk Factors for Mortality and Endotracheal Intubation after Methadone Intoxication. **. Wrong study type. Focus on single drug.**

23. Heier E, et al. Clinical effect of ethanol co-use in patients with acute drug toxicity involving the use of central nervous system depressant recreational drugs.: **Wrong outcome.**

24. Hodgkinson DW, et al. A review of the management of oral drug overdose in the Accident and Emergency Department of the Royal Brisbane Hospital. **Wrong outcome.**

25. Hori S, et al. Clinical characteristics of patients who overdose on multiple psychotropic drugs in Tokyo. **Wrong study type. Focused on single drug.**

26. Irvin CB, et al. Should trauma patients with a Glasgow Coma Scale score of 3 be intubated prior to hospital arrival? **Wrong population. Trauma patients.**

27. Jung S, et al. Interaction between γ-Hydroxybutyric Acid and Ethanol: A Review from Toxicokinetic and Toxicodynamic Perspectives. **Wrong study type. Review article.**

28. Lapatto-Reiniluoto O, et al. A prospective study of acute poisonings in Finnish hospital patients. **Wrong outcome.**

29. Lashin H, et al. Evaluation of various scoring systems as predictors of the need for intensive care unit admission and other adverse outcomes among patients with acute clozapine poisoning. **Wrong study type. Focused on single drug.**

30. Martín-González F, et al. Effectiveness and predictors of failure of noninvasive mechanical ventilation in acute respiratory failure. **Wrong population.** **Respiratory failure patients.**

31. Mizu D, et al. Etiology and clinical characteristics of patients with severely impaired consciousness in prehospital settings: A retrospective study. **Wrong setting. Pre-hospital.**

32. Nemati K, et al. Comparative Study of Demographic and Toxico-Clinical Factors of Patients with Acute Poisoning Admitted to General Intensive Care Unit versus Specific Intensive Care Unit for Poisoning Cases. **Wrong outcome.**

33. Pellatt R, et al. Intubation for patients with overdose: Time to move on from the Glasgow Coma Scale. **Wrong study type. Opinion piece.**

34. Rezar R, et al. Management of intoxicated patients - a descriptive outcome analysis of 4,267 ICU patients. **Wrong setting. ICU. Not about airway management.**

35. Sauter TC, et al. Intubation in acute alcohol intoxications at the emergency department. **Wrong study type. Focused on single drug.**

36. Shastry S, et al. Management of patients with acute drug overdose in an emergency department observation unit. **Wrong study type. Secondary analysis of data.**

37. Stockham P, et al. Characteristics of analytically confirmed gamma-hydroxybutyrate (GHB) positive patients in the emergency department: presentation, poly-drug use, disposition and impact on intensive care resource utilisation. **Wrong outcome.**

Wrong study type - 16

Wrong outcome - 9

Wrong setting - 4

Wrong population - 7

Wrong exposure - 1
